# Altered dynamical integration/segregation balance during anesthesia-induced loss of consciousness

**DOI:** 10.3389/fnetp.2023.1279646

**Published:** 2023-12-05

**Authors:** Louis-David Lord, Timoteo Carletti, Henrique Fernandes, Federico E. Turkheimer, Paul Expert

**Affiliations:** ^1^ Department of Psychiatry, University of Oxford, Oxford, United Kingdom; ^2^ Institut Méditerranéen de Recherches Avancées (IMéRA), Aix-Marseille Université, Marseille, France; ^3^ Department of Mathematics and Namur Institute for Complex Systems (naXys), University of Namur, Namur, Belgium; ^4^ Centre for Music in the Brain, Department of Clinical Medicine, Aarhus University, Aarhus, Denmark; ^5^ Department of Neuroimaging, Institute of Psychiatry, Psychology and Neuroscience, King’s College London, London, United Kingdom; ^6^ Global Business School for Health, University College London, London, United Kingdom

**Keywords:** neural synchronisation, ECoG, altered state of consciousness, integration/segregation, anesthesia

## Abstract

In recent years, brain imaging studies have begun to shed light on the neural correlates of physiologically-reversible altered states of consciousness such as deep sleep, anesthesia, and psychedelic experiences. The emerging consensus is that normal waking consciousness requires the exploration of a dynamical repertoire enabling both global integration i.e., long-distance interactions between brain regions, and segregation, i.e., local processing in functionally specialized clusters. Altered states of consciousness have notably been characterized by a tipping of the integration/segregation balance away from this equilibrium. Historically, functional MRI (fMRI) has been the modality of choice for such investigations. However, fMRI does not enable characterization of the integration/segregation balance at sub-second temporal resolution. Here, we investigated global brain spatiotemporal patterns in electrocorticography (ECoG) data of a monkey (*Macaca fuscata*) under either ketamine or propofol general anesthesia. We first studied the effects of these anesthetics from the perspective of band-specific synchronization across the entire ECoG array, treating individual channels as oscillators. We further aimed to determine whether synchrony within spatially localized clusters of oscillators was differently affected by the drugs in comparison to synchronization over spatially distributed subsets of ECoG channels, thereby quantifying changes in integration/segregation balance on physiologically-relevant time scales. The findings reflect global brain dynamics characterized by a loss of long-range integration in multiple frequency bands under both ketamine and propofol anesthesia, most pronounced in the beta (13–30 Hz) and low-gamma bands (30–80 Hz), and with strongly preserved local synchrony in all bands.

## 1 Introduction

Brain activity is characterized by the dynamic exploration of a diverse and flexible repertoire of functional brain configurations over time (Kelso, 2012; Allen et al., 2014). These explorations of the brain’s repertoire notably enable both global integration, i.e., long-distance interactions between brain regions, and local processing in functionally specialized clusters, i.e., segregation (Sporns, 2013; Deco et al., 2015; Lord et al., 2017). The simultaneous occurrence of integration and segregation, a hallmark of complex systems displaying emerging properties (Turkheimer et al., 2019; Turkheimer et al., 2021), is thought to lead to activity patterns of sufficiently high neural complexity to underlie normal, waking consciousness (Tononi and Edelman, 1998; Cavanna et al., 2018).

It follows that events, be they pathological or pharmacological, which disrupt the integration/segregation balance should in turn modulate one’s behavior and/or subjective experience. Notably, functional MRI (fMRI) dynamic connectivity has been investigated in different sleep stages, pharmacologically induced anesthesia in humans and during psychedelic experiences (Damaraju et al., 2020; Girn et al., 2023). The emerging consensus is that unconscious states are associated with a loss of functional integration, as dynamic explorations become limited to specific patterns dominated by rigid functional configurations tied to the anatomical connectivity (Tagliazucchi et al., 2013; Cavanna et al., 2018; Demertzi et al., 2019; Luppi et al., 2020; Luppi et al., 2021a). On the other hand, psychedelic experiences, such as those induced by taking LSD or psilocybin, have been associated with increased global integration and dynamical explorations loosely constrained by the structural connectome (Carhart-Harris et al., 2014; Petri et al., 2014; Atasoy et al., 2017; Lord et al., 2019; Luppi et al., 2021b). It is however important to note that, because fMRI allows for brain activity measurements on the order of seconds, the aforementioned studies have not yet determined how the integration/segregation balance is modulated on a sub-second scale in physiologically-reversible states of altered consciousness, such as general anesthesia.

Here, we investigated global brain spatiotemporal patterns in electrocorticography (ECoG) data of monkeys under either ketamine or propofol general anesthesia from the publicly available NeuroTycho project (Yanagawa et al., 2013). While administration of both ketamine and propofol induces a rapid decrease in level of consciousness, the respective pharmacodynamics of these drugs markedly differs. Ketamine acts primarily as a NMDA receptor antagonist whereas propofol’s main target is the GABAA receptor on which it has a positive allosteric effect (Brown et al., 2011; Stevens, 2022). ECoG is an invasive imaging modality in which an electrode array is applied directly on the cortical surface. This allows for brain activity measurements on significantly faster timescales than fMRI. For example, synchronization in the beta (13–30 Hz) and low gamma (∼30–80 Hz) frequency bands between neuronal populations is thought play a crucial role in the binding of information into conscious representations (Kopell et al., 2000; Varela et al., 2001; Tallon-Baudry, 2009; Betti et al., 2021) and evidence shows it acts as a substrate for the emergence of functional networks (Lord et al., 2013a). These links between high-frequency electrical oscillations and higher level cognitive entities cannot be directly investigated with fMRI due to insufficient temporal resolution.

In the present study, we first investigated the effects of ketamine and propofol from the perspective of band-specific synchronization behavior across the entire ECoG array, analyzing the collective behavior of ECoG channels treated as oscillators. We further sought to determine whether synchrony within spatially localized clusters of oscillators was differently affected by ketamine and propofol in comparison to synchronization over spatially distributed subsets of ECoG channels. This analysis hence enabled a quantification of the dynamics underlying the integration/segregation balance under anesthesia on physiologically-relevant timescales. The findings reflect global brain dynamics characterized by a loss of global integration in multiple frequency bands under both ketamine and propofol anesthesia, most pronounced in the beta (13–30 Hz) and low-gamma bands (30–80 Hz). Furthermore, whilst long-range synchronization was reduced during anesthesia relative to wakeful rest in those same frequency bands, synchrony within spatially localized electrode clusters remained strongly conserved for both anesthetic agents. Thus, despite their distinct pharmacodynamics, the effects of ketamine and propofol strongly converge on the novel measures of neural synchronization here applied to ECoG data.

## 2 Methods

### 2.1 Electrocorticography data acquisition and preprocessing

Neuroimaging data were obtained from the NeuroTycho dataset, an open-source set of 128-channel invasive electrocorticography (ECoG) recordings in a *macaca fuscata* monkey collected at the Riken Institute (Tokyo, Japan) (Yanagawa et al., 2013). The ECoG array covered the left hemisphere only, with electrodes placed on both the lateral and medial brain surfaces. Data were recorded during both a resting-state baseline with eyes closed and during either propofol or ketamine-induced anesthesia in separate experiment. Each experiment was carried out least 4 days apart. During each experiment, the monkey was seated in a primate chair with both arms and head movement restricted. The “anesthesia” condition was considered to have begun once the monkey failed to respond to sensory stimulation (i.e., stimulation of the nose with a cotton swab and unresponsiveness to have their forepaw touched). In the propofol condition, the monkey was given a single bolus of intravenous propofol (5.2 mg/kg), until loss of consciousness was observed. For the ketamine condition, a single bolus of intramuscular ketamine (5.1 mg/kg) was administered. For either drug, no maintenance anesthesia was given following initial induction.

ECoG data were acquired at a 1,000 Hz sampling rate. As is common practice in electrophysiological studies, the quality of ECoG signals was assessed by visually inspecting the power spectra of individual ECoG channels, and no channels were discarded on this basis. The data were notch filtered at 50 Hz to account for electrical line noise in Japan. While we did not downsample the data, we re-binned the timecourses into 100 ms windows in order for our measures of inter-areal synchrony (see sections below) to operate on physiologically meaningful timescales. To ensure a consistent number of time points across all experimental conditions (ketamine/propofol and baseline), we considered the first 5,000 time points of each experimental run, which yields a 5,000*100 ms = 500 s (8.33 min) recording interval.

### 2.2 Calculation of band-specific phase angles

We bandpassed each ECoG channel timecourse for the frequency-band of interest: δ = 0–4 Hz; θ = 4–8 Hz; α = 8–13 Hz; β = 13–30 Hz, and γ = 30–80 Hz. The bandpassed filtered data were normalized by removing the mean of each channel and dividing it by its standard deviation. For each ECoG channel n, we compute its analytic signal $x_{a}^{n}\left(t\right)=x^{n}\left(t\right)+iH\left(x^{n}\left(t\right)\right)$ by using the Hilbert transform $H\left(x\left(t\right)\right)$ . We then extract the instantaneous phase $\theta^{n}\left(t\right)=\arg\;\left(x_{a}^{n}\left(t\right)\right)$ . All analyses were performed using Matlab (MathWorks, Inc.).

**FIGURE 1:**
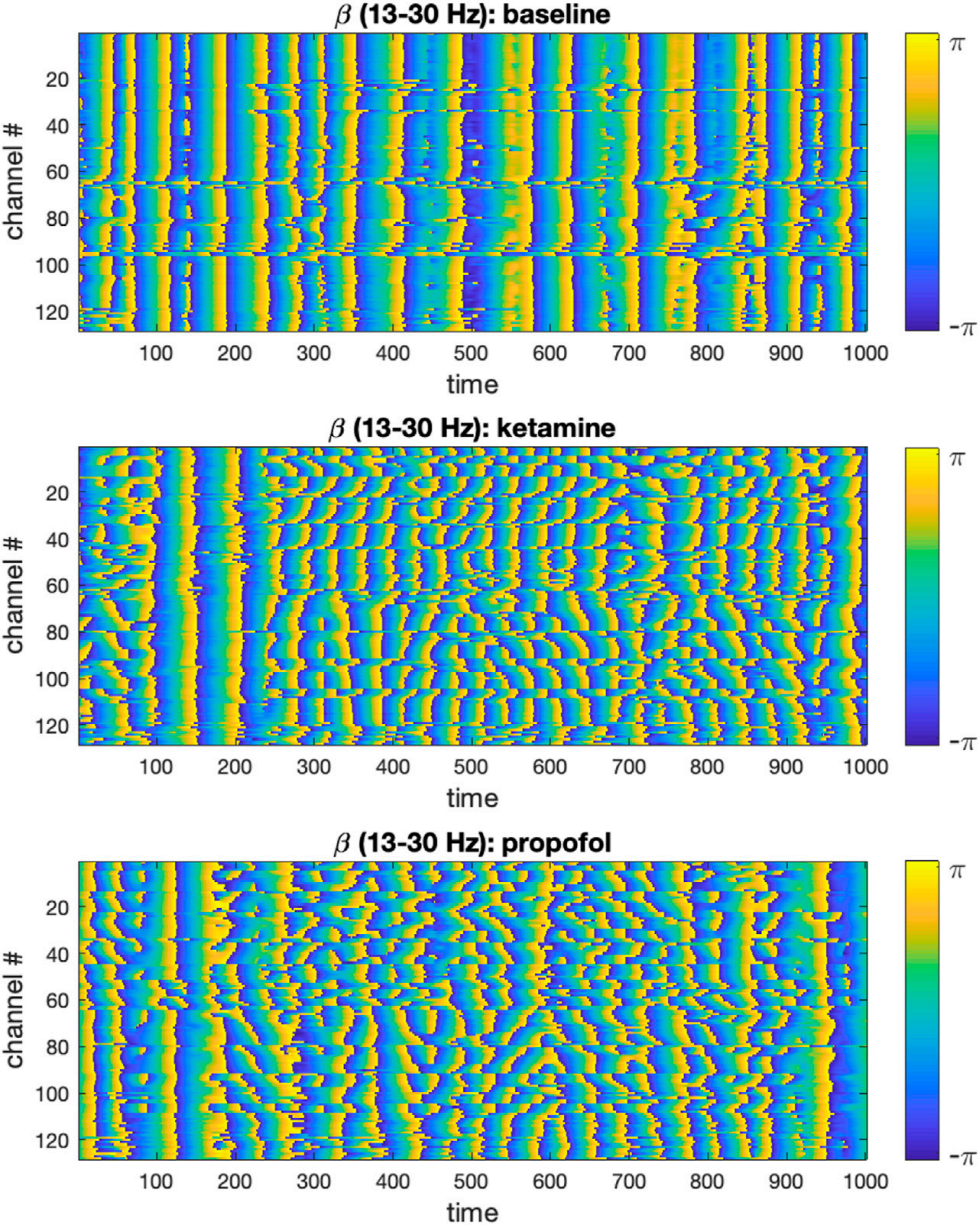
Time-evolution of phase angles θ derived from the ECoG signal via Hilbert transformation in the β-band is plotted for 1,000 time steps (binned 100 ms intervals) in the baseline, ketamine and propofol states. It can be qualitatively observed that for both drugs, the phase angles are on average less aligned at any given time point. We only show the β -band here for concision and to illustrate the methodology, but this same analysis step was performed for the other frequency bands under study (δ, α, γ) to enable statistical comparison (using all 5,000 time steps).

We visualized the time-evolution of the phase angles for each ECoG channel in different frequency bands for both experimental conditions. For visualization purposes, only the first 500 time steps were plotted. To illustrate this output, the time-evolution of phase angles $\theta^{n}\left(t\right)$ for ketamine, propofol and the respective wakeful rest baseline are shown in Figure 1 for the β-band (13–30 Hz):

### 2.3 Analysis of global synchronization of ECoG channels

For each of the aforementioned frequency bands, we computed the order parameter (OP) (Kuramoto, 1975) of the whole set of recording electrodes (*n* = 128 ECoG channels) at each time point (t = 5,000):

$$OP\left(t\right)=\frac{1}{N}\left|\sum_{n=1}^{N}e^{i\theta_{n}\left(t\right)}\right|$$

This measure enables us to quantify the extent of the global desynchronization induced by ketamine and propofol anesthesia relative to the wakeful rest baseline. The time-evolution of the OP in each frequency band for the anesthesia and baseline conditions were plotted, see Figure 4. Differences in the average value of the OP were compared between the baseline and anesthesia conditions by using paired t-tests (undirected) with Bonferroni correction for multiple comparisons.

### 2.4 Comparison of local vs. distributed cluster synchrony

**FIGURE 2:**
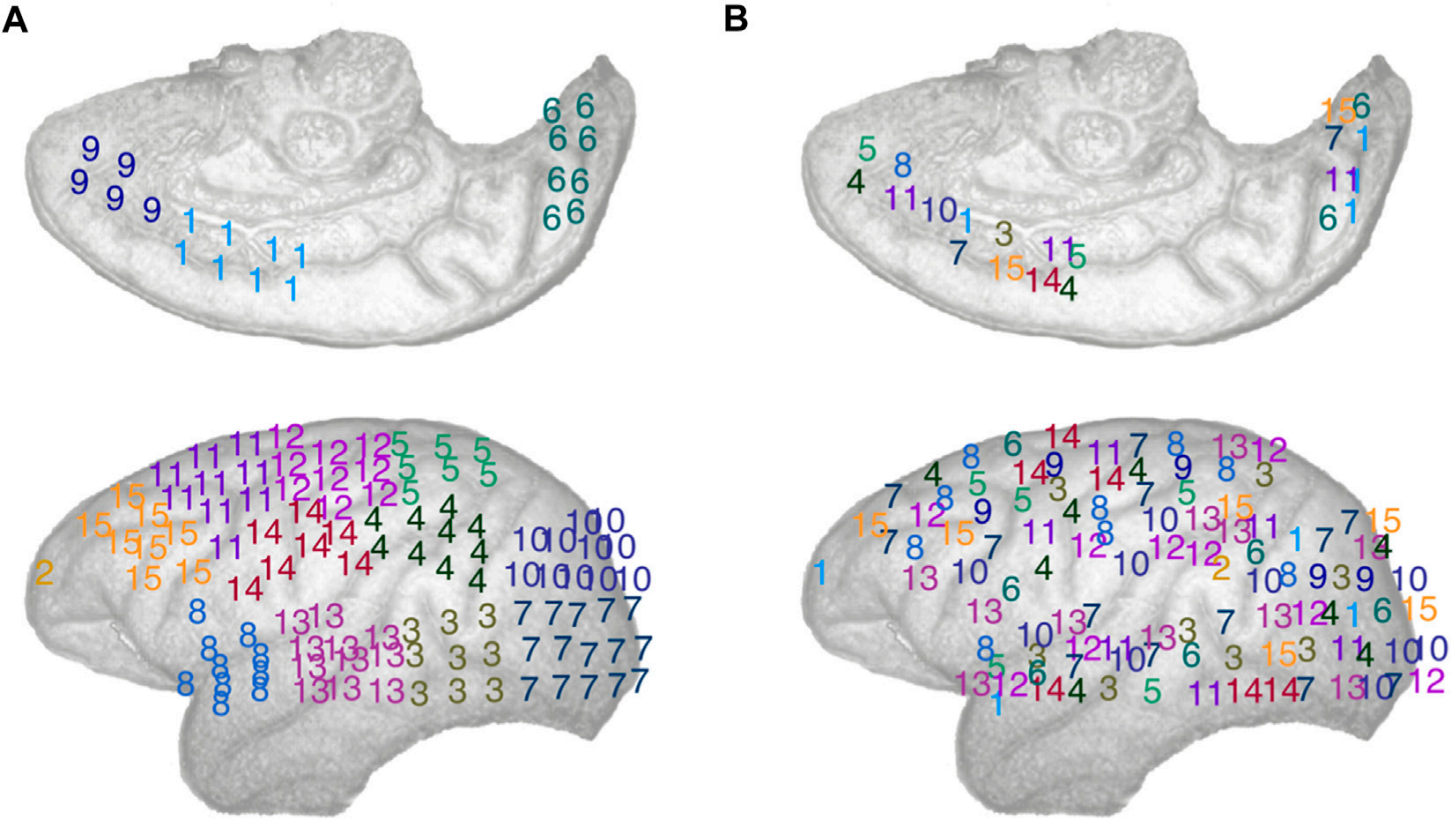
Schematic representation of the electrodes array implemented in the left cortical surface of the monkey, inside surface on the top row, outside surface on the bottom row. The electrodes are numbered according to: **(A)** A typical spatially local clustering for the electrodes using k-means clustering with k = 14. **(B)** A typical spatially distributed clustering from random electrodes assignment for k = 14. See Methods: Comparison of local vs. distributed cluster synchrony for details.

We then shifted our attention to studying the brain dynamics from the perspective of clusters of electrodes seen as oscillators. To do so, we applied k-means clustering, using the Matlab kmeans function, to obtain clusters of ECoG channels. K-means is a widely used unsupervised clustering method that clusters elements of a set based on the similarity of their features into k clusters (MacQueen, 1967), In our case, we clustered the electrodes using their spatial coordinates as features, effectively clustering together spatially local channels, for: k = 13, 14, 15. These k-values ensure that the number of electrodes per cluster is small enough to obtain well localized clusters, compared to the size of the brain area mapped by the electrodes, that also contain a non-trivial number of electrodes. To deal with the latter point, we introduced a constraint in the k-mean algorithm so that the smallest possible number of electrodes in a given cluster was *n* = 4. To account for the randomness involved in k-means initialization and ensure replicability of our findings, we performed 100 iterations of the k-means algorithm in our analyses. A typical exemplar of community assignments obtained for k = 14 is shown in Figure 2A. Where electrodes are assigned a number (and colour) that indicate which cluster they belong to.

For each cluster of electrodes C, we calculated the internal synchrony $\varphi_{C}$ at each time step *t*:

$$\varphi_{C}\left(t\right)=\frac{1}{\left|C\right|}\left|\sum_{r\in C}e^{i\theta_{r}\left(t\right)}\right|$$

where |C| denotes the size of the cluster under scrutiny. Local synchrony was calculated for each frequency band on the clusters of ECoG channels defined by the k-means clustering algorithm, i.e., spatially compact clusters, for all k values (k = 13, 14, 15). This process was repeated for each of the 100 k-means iterations.

**FIGURE 3:**
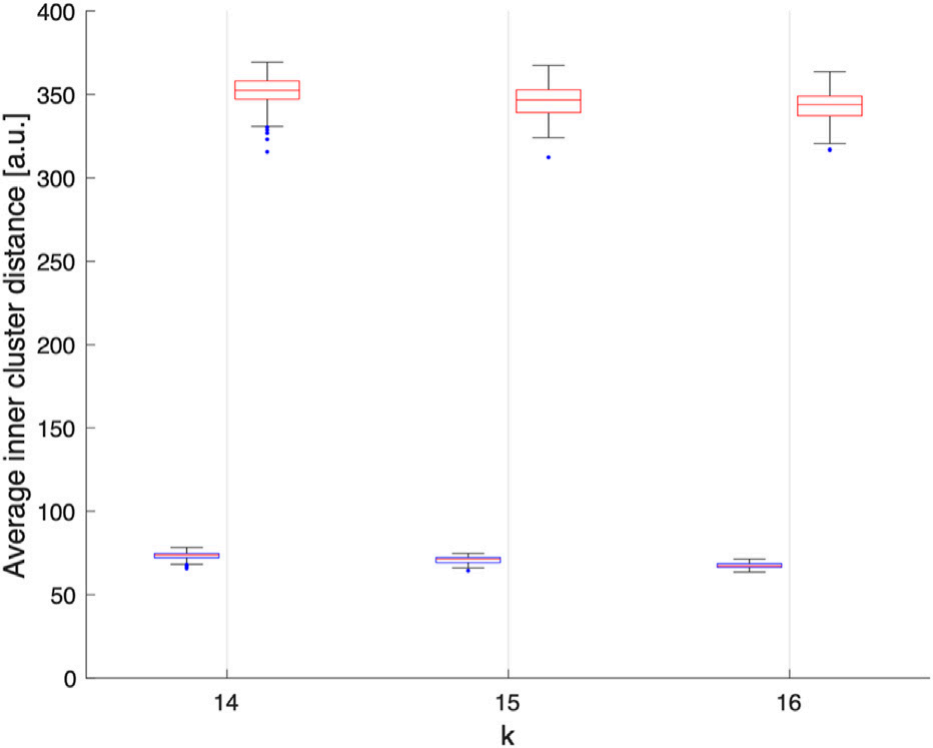
Average Euclidean distance, in arbitrary units, between electrodes within each clusters obtained assuming k = 14, 15 or 16 over 100 realisations. We can observe that electrodes grouped by k-means clustering are spatially compact (blue), while the electrodes grouped randomly are spatially delocalized (red).

To measure synchrony across non spatially compact cluster of electrodes, i.e., distributed synchrony, we randomly assigned electrode to k clusters, thus creating spatially distributed clusters of electrodes, see Figure 2B for an illustration. We can see that the numbers (and colours), representing the cluster an electrode belongs to, are effectively spatially distributed. To ensure the robustness of this approach over a large number of randomly assigned electrode clusters, we computed local synchrony on 100 distinct sets of distributed clusters for k = 13, 14, and 15 respectively. For consistency, we introduced the same constraint as with the k-means, ensuring that the minimum size of any distributed cluster was *n* = 4 ECoG channels. As expected, randomly assigning the electrodes to k clusters statistically significantly increased the average Euclidian distance between electrodes in a given cluster compared to the corresponding k-means algorithm output, thus yielding spatially distributed cluster. See Figure 2 for a typical local and distributed electrodes and Figure 3 for the statistics on the spatial localization of the local and distributed clusters.

For each frequency band and cluster number k, we compared the effects of ketamine and propofol anesthesia on the internal synchrony of local vs. distributed electrode clusters using repeated paired t-tests with Bonferroni correction for multiple comparisons. Thus, the statistical significance of pre vs. post-drug effect on cluster synchrony were separately calculated for 100 local cluster assignments, and 100 distributed cluster assignments respectively using a Bonferroni-adjusted significance threshold of α = 5*10-4.

## 3 Results

### 3.1 Ketamine and propofol induce global, frequency band-specific desynchronization of brain oscillations

**FIGURE 4:**
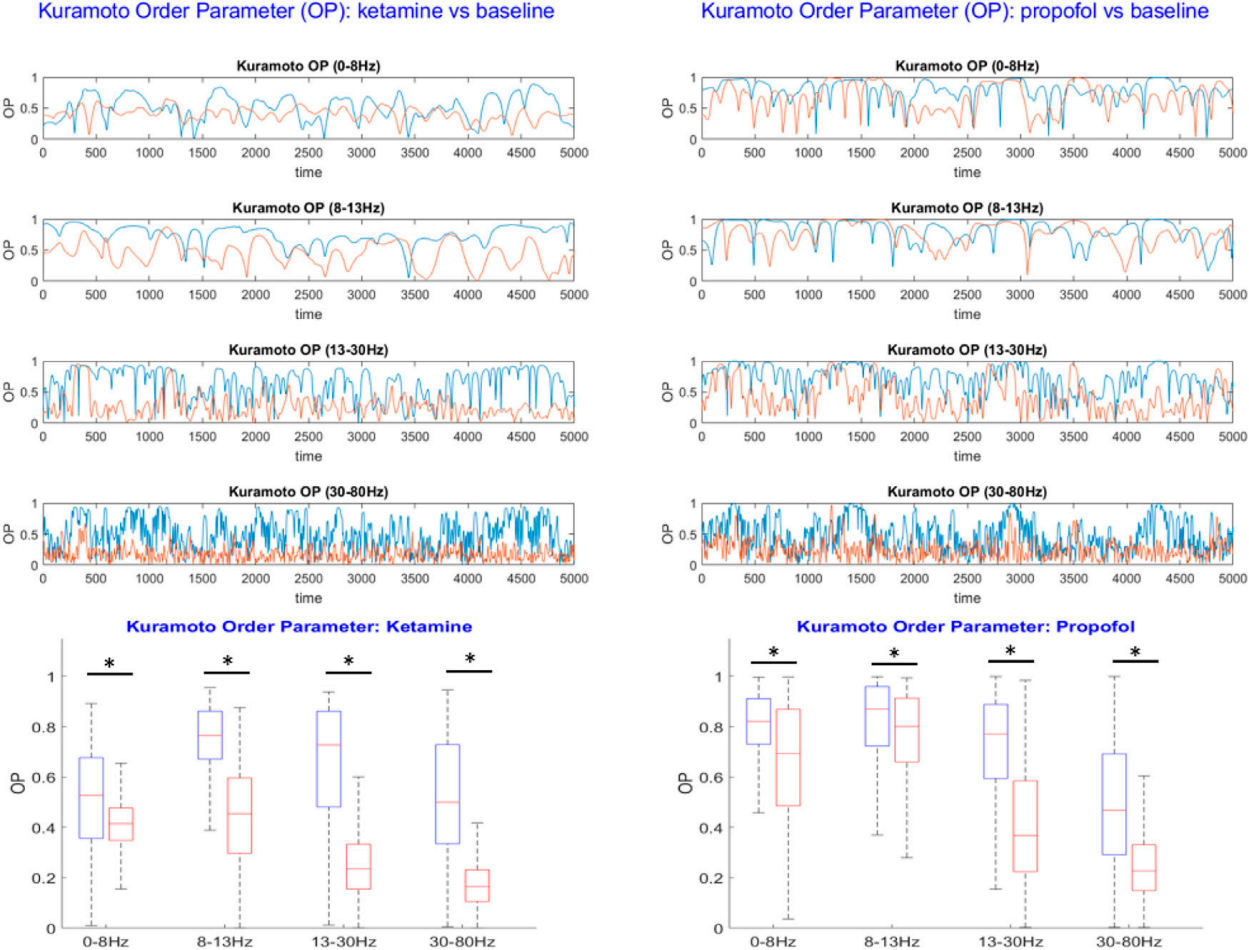
Top: The Kuramoto order parameter (OP) is plotted against time for both the anesthesia condition (red) and the wakeful rest baseline (blue) in each frequency band under study. Results from the ketamine anesthesia are shown on the lefthand side, and results for the propofol anesthesia on the right. Both ketamine and propofol induce a strong and statistically significant desynchronization of brain activity across the entire array of ECoG channels, persistent over all frequency bands under study. The largest effects of anesthetics on global synchrony were observed in the β (13–30 Hz) and γ (30–80 Hz) frequency bands, respectively. Bottom: Boxplots showing the mean OP over time ± standard deviation for the baseline (blue) and anesthesia conditions (red). Statistically significant reductions in global synchrony were observed across all frequency bands for both ketamine (left) and propofol (right). The largest effects on global synchrony were observed in the β (13–30 Hz) and γ (30–80 Hz) frequency bands.

In each frequency band under study, we find that both ketamine and propofol induce a global desynchronization of brain activity across the entire array of ECoG channels, as indicated by significant decreases in the Kuramoto order parameter (OP) of the system in all frequency bands considered, see Figure 4. For ketamine, mean percent decreases in OP during anesthesia relative to the wakeful rest baseline were: −21.3% (0–8 Hz); −40.9% (8–13 Hz); −59.1% (13–30 Hz); −65.6% (30–80 Hz) (all p-vals are sufficiently small to be considered as vanishing numerically). For propofol, mean percent decreases in OP during anesthesia relative to the wakeful rest baseline were: −16.7% (0–8 Hz); −5.9% (8–13 Hz); −41.8% (13–30 Hz); −49.1% (30–80 Hz) (all p-vals are sufficiently small to be considered as vanishing numerically). We note that for both drugs, the largest effects on global synchrony were observed in the β (13–30 Hz) and γ (30–80 Hz) frequency bands. Whilst still statistically significant, the weakest effect was observed for propofol in the α band (8–13 Hz).

### 3.2 Differential effects of general anesthetics on synchronization spatially localized vs. distributed electrode clusters

**FIGURE 5:**
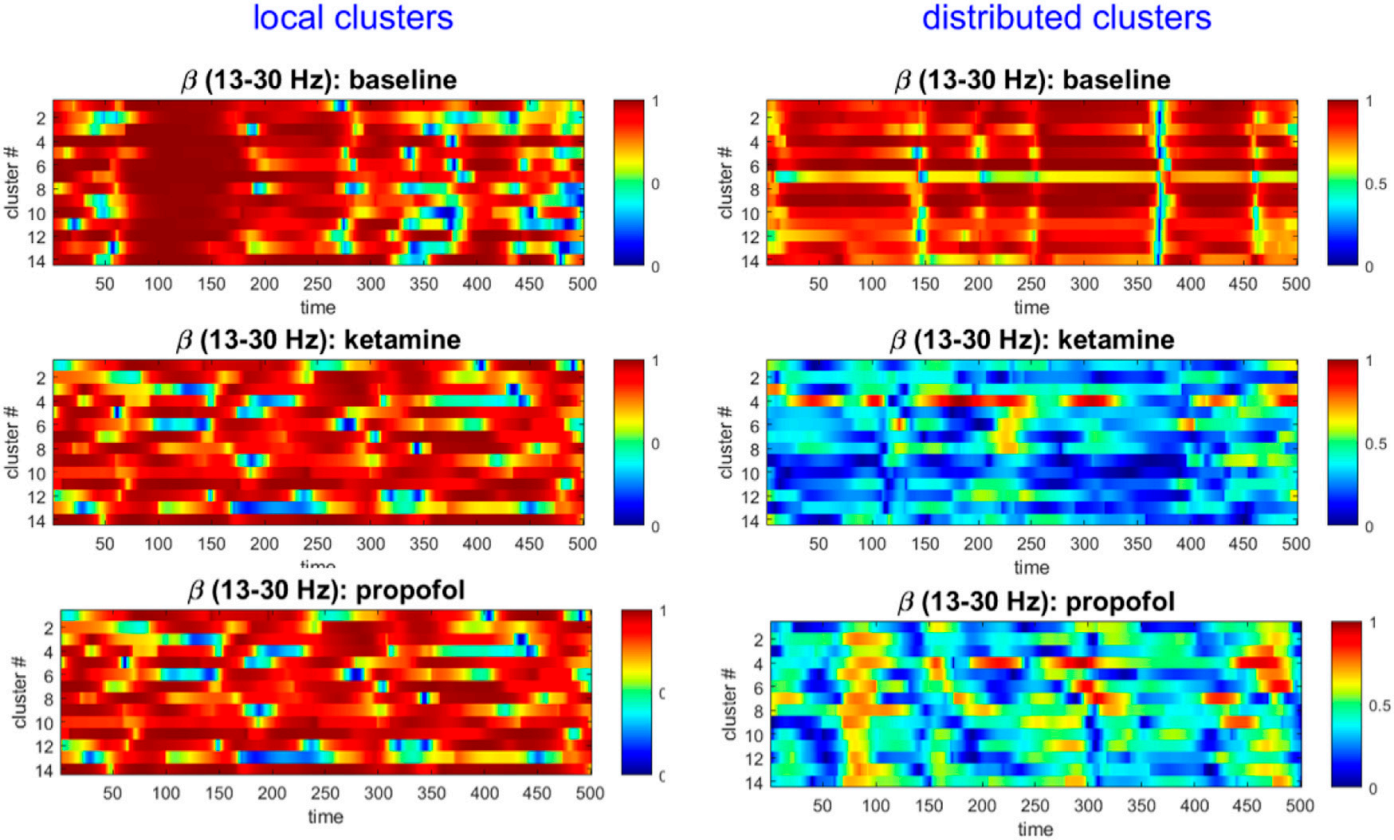
The local cluster synchrony $\varphi_{C}$ is plotted against time for the first 500 time-steps (binned 100 ms intervals) in the baseline, ketamine and propofol states for k = 14 clusters that are either spatially localized (lefthand side) or spatially distributed (righthand side). We only show results from the clusters derived from the β -band here for concision and to illustrate the methodology, but this same analysis was performed for each frequency band under the study to enable statistical comparison (using all 5,000 timesteps). Local cluster synchrony in β-band is preserved under both ketamine and propofol anesthesia. By contrast, distributed cluster synchrony is strongly decreased by each drug.

Because the OP considers the synchronization behavior of the entire electrode array, it does not allow to infer any distinction between local and distributed effects of the drugs on brain dynamics. We therefore followed up the initial analysis by comparing synchronization behavior of spatially localized and spatially distributed clusters of electrodes; the latter requiring long-range synchronization while the former does not. The internal synchrony, i.e., the local OP, of a given cluster at a given time step is denoted as $\varphi_{C}\left(t\right)$ . We provide a visualization of this analysis in Figure 5 below; changes the time-evolution of $\varphi_{C}$ are plotted for 14 clusters derived from beta-band activity (13–30 Hz) under the different experimental conditions. A marked reduction in $\varphi_{C}$ is observed in the distributed clusters of electrodes for both ketamine and propofol anesthesia, but $\varphi_{C}$ remains markedly unchanged between the baseline and anesthesia conditions for the spatially localized clusters.

**FIGURE 6:**
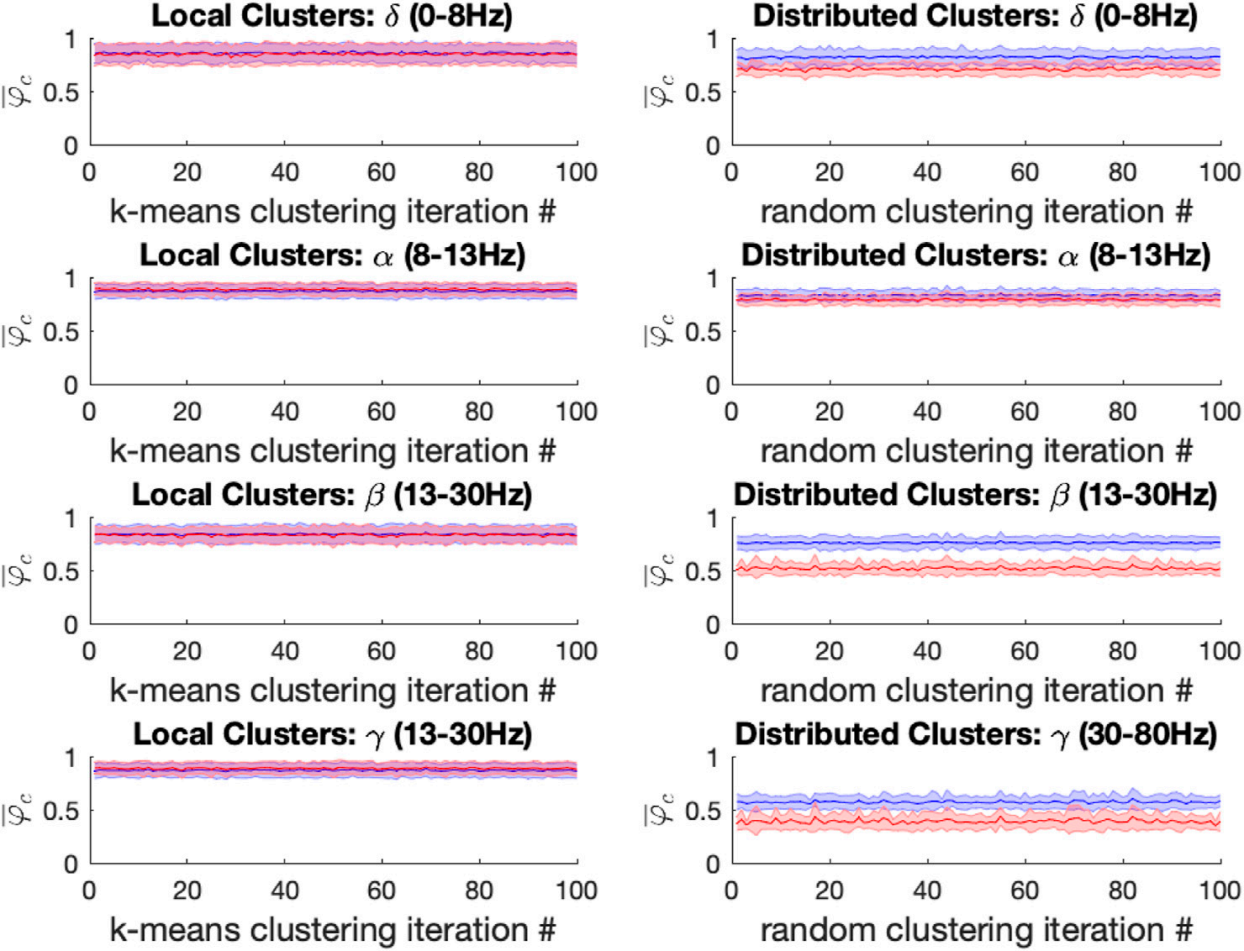
Left: For the ketamine (red) vs. baseline (blue) conditions, the mean local cluster synchrony ${\bar{\varphi }}_{C}$ ± standard deviation is plotted for each of 100 k-means iterations (x-axis) in each of the four frequency bands under study for k = 14. Repeated within-condition t-tests failed to reach statistical significance at the Bonferroni-corrected threshold (α = 5*10-4) in any of the four frequency bands of interest. Right: Mean distributed cluster synchrony ${\bar{\varphi }}_{C}$ ± standard deviation is plotted for each of 100 iterations of the pseudo-random algorithm used for distributed cluster assignments (x-axis). Statistically significant reductions in distributed cluster synchrony at the Bonferroni-corrected significance threshold (α = 5*10-4) were observed following ketamine anesthesia for all (100/100) distributed cluster assignments in the α, β and γ bands, respectively. By contrast no significant differences in drug effects on distributed cluster synchrony were observed in the δ-band.

**FIGURE 7:**
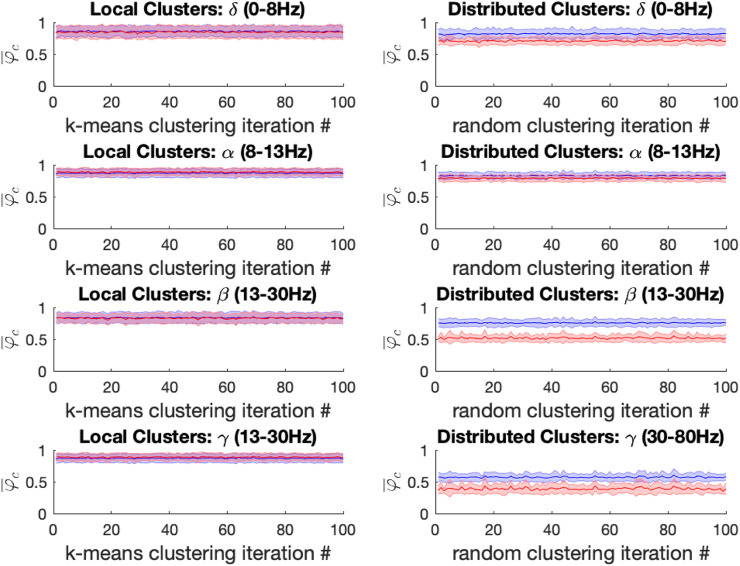
Left: For the propofol (red) vs. baseline (blue) conditions, the mean local cluster synchrony ${\bar{\varphi }}_{C}$ ± standard deviation is plotted for each of 100 k-means iterations (x-axis) in each of the four frequency bands under study for k = 14. Repeated within-condition t-tests failed to reach statistical significance at the Bonferroni-corrected threshold (α = 5*10-4) in any of the four frequency bands of interest. Right: Mean distributed cluster synchrony ${\bar{\varphi }}_{C}$ ± standard deviation is plotted for each of 100 iterations of the pseudo-random algorithm used for distributed cluster assignments (x-axis). Statistically significant reductions in distributed cluster synchrony at the Bonferroni-corrected significance threshold (α = 5*10-4) were observed following propofol anesthesia for all (100/100) distributed cluster assignments in the β and γ bands, respectively. By contrast only 6/100 significant differences in propofol’s effect on distributed cluster synchrony were observed in the α-band, while more mixed results were observed in the δ-band with 60/100 of distributed cluster assignments reaching significance.

For both ketamine and propofol, synchrony was strongly maintained in spatially localized electrode clusters of ECoG channels, defined by k-means clustering; see Methods section, for k = 14. For ketamine, synchronization in spatially localized clusters was not significantly different in any of the frequency bands at the Bonferroni-adjusted significance threshold (α = 5*10-4). The average *p*-values were: 0.618, 0.010, 0.019, and 0.383 across all 100 k-means iterations for the δ, α, β, and γ bands, respectively, see Figure 6 left column. Similarly, for propofol anesthesia, synchronization within local clusters was also preserved relative to baseline in all frequency bands and across all cluster assignments. The averaged *p*-values, obtained across all 100 k-means iterations, were: 0.77, 0.28, 0.74, and 0.76 for δ, α, β, and γ frequency bands, see Figure 7 left column.

Under ketamine anesthesia, synchronization in spatially distributed clusters was strongly and significantly reduced in every distributed cluster assignment (100/100) for the α, β, and γ bands, but no such reductions were observed in the δ band (0/100), see Figure 6 right column. The average p-vals were: 0.09, 3*10-7, 8*10-7, and 3*10-6 for δ, α, β, and γ frequency bands respectively (Bonferroni-adjusted significance threshold α = 5*10-4). Under propofol anesthesia distributed cluster synchrony was significantly reduced for every cluster assignment (100/100) in the β, and γ bands with average *p*-values of 1.1*10-7 and 2.7*10-5 respectively, see Figure 7 right column. Statistically significant reductions in distributed cluster synchrony were observed for 60/100 cluster assignments in the δ band (average p-val = 0.0012) but for only 6/100 cluster assignments in the α band (average p-val = 0.017).

Thus, for both ketamine and propofol, statistically significant reductions in distributed cluster synchrony, that are robust over cluster assignments, were observed in the β, and γ frequency bands. By contrast, synchronization in local clusters was not significantly impacted by either drug.

## 4 Discussion

The integration/segregation balance is intrinsically linked to some of the most complex and fascinating phenomena of modern neuroscience; from the spontaneous emergence of resting-state networks to higher cognition and consciousness (Varela et al., 2001; Sporns, 2013; Lord et al., 2017; Luppi et al., 2021a). Yet, relatively little is known about how changes in subjective experience relate to time-resolved changes in the integration/segregation balance. Here we applied novel analytical approaches to characterize changes in frequency band-specific synchronization patterns between clusters of ECoG channels in a monkey under general anesthesia, compared to a wakeful rest baseline. The effects of both ketamine and propofol anesthesia were studied across separate scanning sessions. We placed specific emphasis on investigating the dynamical integration/segregation balance during anesthesia-induced loss of consciousness by considering individual ECoG channels as oscillators.

Our findings describe a brain functional architecture characterized by a loss of global functional integration during both ketamine and propofol-induced loss of consciousness caused by a selective disruption in long-range synchrony, particularly in the β and γ frequency bands. On the other hand, synchronization in spatially localized clusters remained unaffected by both anesthetic drugs relative to a wakeful rest baseline in all frequencies under study. Furthermore, the results were highly consistent upon building electrode clusters of different sizes (k = 13, 14, 15) (see Supplementary Materials). Thus, despite their markedly different pharmacodynamics (Brown et al., 2011; Stevens, 2022), both ketamine and propofol had significant and directionally consistent effects on the synchronization measures employed in this study at general anesthetic doses, especially in the β and γ frequency bands.

The formation of dynamic links between brain regions via synchronization of oscillations in different frequency bands is one of the most plausible mechanisms for large-scale integration in the brain, and for the resulting binding of information into unified cognitive moments (Joliot et al., 1994; Varela et al., 2001; Tallon-Baudry, 2009; Lord et al., 2013a). For both ketamine and propofol anesthesia, we found that long-range synchronization was most strongly disrupted in the beta (13–30 Hz) and gamma (30–80 Hz) frequency bands. Interestingly, oscillations in the beta frequency range have been shown to facilitate long-range interactions at the cortical level (Gross et al., 2004; Sehatpour et al., 2008; Tallon-Baudry, 2009). Similarly, several lines of evidence have implicated gamma-oscillations in both long-range synchronization and as a neural correlate of conscious perception as well as sensory information-guided, goal-directed behaviors (Kopell et al., 2000; Varela et al., 2001; Melloni et al., 2007; Chand and Dhamala, 2017). Notably, the strength of synchronization in β and γ bands in large-scale cortical networks predicts the perception of audiovisual stimuli, further highlighting the important role of these frequency bands for cross-modal integration (Hipp et al., 2011). Thus, our findings of strongly reduced long-range synchrony in these particular frequencies during anesthesia-induced loss of consciousness provides further evidence for the contribution of the beta and gamma bands toward the integration of information into conscious representations.

Whilst detailed mechanistic explanations linking the pharmacodynamics of propofol and ketamine general anesthesia to the network effects reported in this study is beyond the scope of the current investigation, we nonetheless wish to expand on this idea. Binding of propofol to the widely distributed GABAA receptor hyperpolarizes pyramidal neurons, disrupting the balance between excitatory-to-inhibitory inputs in favor of inhibitory drive (Breshears et al., 2010; Brown et al., 2011). On the other hand, experimental evidence suggests that ketamine binds preferentially to NMDA receptors on GABAergic inhibitory interneurons, thereby disinhibiting pyramidal neurons and leading to a dissociative state in which multiple brain areas communicate through aberrant activity that lacks normal spatial and temporal coordination (Seamans, 2008; Brown et al., 2011). Thus, the most likely shared mechanism between those two drugs is an alteration of the ratio between excitation and inhibition (E/I ratio) in pyramidal neurons. Efficient reciprocal connections between fast-spiking inhibitory interneurons and pyramidal neurons is a crucial requirement for giving rise to coordinated, high-frequency oscillations in distributed circuits (Lord et al., 2013b). Notably, inhibition of fast-spiking interneurons using optogenetic techniques selectively suppresses γ-oscillations (Sohal et al., 2009). Moreover, NMDA receptor deletion results in a loss of neuronal synchronization in high frequency bands *in vitro* (Belforte et al., 2010). Therefore, we suggest that both ketamine and propofol alter the E/I ratio in pyramidal neurons, which may lead to significant disruptions in long-range synchronization in the β and γ bands, as observed in this study.

Whilst the focus of our analysis was on the β and γ frequency bands due to their well-documented roles in conscious perception, as discussed above, we wish to briefly comment on the results from the lower frequency bands under study (i.e., θ and α). We found decreased synchrony between distributed clusters of electrodes in those bands, albeit not as pronounced as in β and γ bands. This is interesting in light of the fact that oscillatory power in lower frequency bands is generally thought to be increased under propofol anesthesia (Ní Mhuircheartaigh et al., 2013). We note that biophysical modelling studies have found alternations between continuous low-frequency band spiking in thalamus and periods of co-occurring thalamic and cortical silence under propofol anesthesia (Soplata et al., 2023). Loss of cortical synchrony in low-frequency bands, and coordinated thalamocortical silent periods may therefore also contribute to the unconscious state; an observation consistent with our results.

While the available experimental data constrained our analysis to ECoG data from a single hemisphere, the available evidence suggests that both ketamine (Bonhomme et al., 2016; Lv et al., 2016) and propofol (Schrouff et al., 2011; Pujol et al., 2021) lead to the desynchronization of functional connectivity across spatially distant brain regions in both hemispheres; as such we would expect our results to remain consistent, and potentially even strengthen, should the analyses have included data from both hemispheres. Moreover, both GABAA receptors (propofol targets) (Bowery et al., 1987) and NMDA receptors (ketamine targets) (Hansen et al., 2017) are widely distributed in the brain, reducing the likelihood of hemisphere-specific effects.

The present results are also in agreement with prior studies which have used dynamical network analyses and related approaches to study physiologically-reversible changes in consciousness. The emerging consensus from this growing body of fMRI literature is that conscious wakefulness is characterized by global integration, (i.e., long-distance interactions between brain regions displaying scaling behaviour in time and space across a range of imaging modalities) (Linkenkaer-Hansen et al., 2001; Expert et al., 2011), as well as by the dynamic exploration of a diverse and flexible repertoire of functional brain configurations (Watanabe et al., 2014; Cabral et al., 2017; Gu et al., 2018). Conversely, during anesthesia or deep sleep, long-range functional integration is reduced and the dynamic explorations become limited to a smaller subset of specific activity patterns, more tightly constrained by the structural connectome (Demertzi et al., 2019). Our results therefore extend the idea of a dynamical imbalance between the integration and segregation of information during altered consciousness to electrophysiological data at much faster timescales than fMRI investigations allow for.

The results presented in this paper are functional and model agnostic. There is however a large body of theoretical work relating brain structure and function (Park and Friston, 2013). In particular, the modular structure of the brain (Meunier et al., 2010) has been shown to play a central role in the integration and segregation of brain function (Wang et al., 2021). Moreover, theoretical work has shown that transmission delays between neural populations (Pariz et al., 2021) or nodes of a Kuramoto model on modular network (Ziaeemehr et al., 2020) do have an important impact on the synchrony between brain regions and particularly in the transition between synchronous and asynchronous states. One might then conjecture that the pharmacodynamic effects of the two drugs considered have a functional effect on the transmission of information between brain regions, effectively playing the role of a global synchronization control parameter (Lynn and Bassett, 2019).

The measures introduced in this study capture distributed changes in the brain’s spatiotemporal organization induced by ketamine and propofol without considering the precise localization these changes within the brain. Specifically, they demonstrate the potential sensitivity of time-resolved measures of system-wide integration/segregation balance to changes in consciousness level and/or contents. We suggest that related measures could be extended beyond anesthesia and remain sensitive to a range of conditions in which behavior and/or subjective experience is measurably altered, i.e., psychopharmacological effects, neuropsychiatric illness, sleep, disorders of consciousness, etc. For example, we would notably expect psychedelic drugs, e.g., psilocybin and LSD, to have opposite effects as general anesthetic drugs on the measures employed in this study, since some fMRI studies have shown that these compounds may actually increase long-range functional integration (Petri et al., 2014; Tagliazucchi et al., 2016; Lord et al., 2019). An extension of this idea is that biomarkers for neurological and psychiatric disorders need not be necessarily based on changes in specific brain areas or circuits, but could also be reflected as altered global spatiotemporal patterns. For example, dynamical functional connectivity analyses of fMRI data from patients with major depressive disorder revealed abnormally stable patterns of long-range synchronization, consistent with various clinical features of depressive patients, including ruminative, slow, and monotonous thinking (Demirtaş et al., 2016; Figueroa et al., 2019). The present work lays a foundation for further studies in this direction.

In conclusion, we investigated changes in the dynamical balance between integration and segregation of information during anesthesia-induced loss of consciousness in ECoG data from a macaque monkey. The analysis was sensitive to the breakdown of global functional integration and long-range synchronization previously documented in fMRI investigations of physiologically reversible unconscious states. We also report that, in contrast to long-range synchronization, functional synchrony in spatially localized clusters of electrodes remained highly conserved under general anesthesia. We therefore propose that time-resolved measures of synchronization behavior in clusters of oscillators may be sensitive to departures from normal waking consciousness in various conditions and be clinically useful.

## Data Availability

Publicly available datasets were analyzed in this study. This data can be found here: http://www.www.neurotycho.org.
