# Supplementary material for: Altered dynamical integration/segregation balance during anesthesia-induced loss of consciousness
Source: Front Netw Physiol. 2023 Dec 5;3:1279646. doi: 10.3389/fnetp.2023.1279646 (PMC10728865; doi:10.3389/fnetp.2023.1279646)
Supplement: Supplementary file 1 [file DataSheet1.PDF]

Supplementary Material for *Altered dynamical integration/segregation balance during anesthesia-induced loss of consciousness*

Louis-David Lord[1,2], Timoteo Carletti[2,3], Henrique Fernandes[1,2,4], Federico E. Turkheimer[5], Paul Expert[2,6]

[1]Department of Psychiatry, University of Oxford, UK

[2] Institut Méditerranéen de Recherches Avancées (IMéRA), Aix-Marseille University, France

[3]Department of Mathematics and Namur Institute for Complex Systems (naXys), University of Namur, Belgium

[4]Centre for Music in the Brain, Department of Clinical Medicine, Aarhus University, Denmark

[5]Department of Neuroimaging, Institute of Psychiatry, Psychology and Neuroscience, King's College London, UK

[6]Global Business School for Health, University College London, UK

*** Correspondence:**
Dr Paul Expert
p.expert@ucl.ac.uk

1. Robustness of results with cluster size

In this supplementary, we report the results of our analysis obtained on clusters of size k=13 and 15 for both Ketamine and Propofol. The results are consistent and robust with the k=14 case in the main text.

Figures *Supplementary 1* and Supplementary 2 show the mean synchrony, with standard deviation, for the baseline and Ketamine conditions in the local cluster (left) and the distributed clusters (right). Figures *Supplementary 3* and *Supplementary 4* show the mean synchrony, with standard deviation, for the baseline and Propofol conditions in the local cluster (left) and the distributed clusters (right). Tables *Supplementary 5* and *Supplementary 6* recapitulate the average p-values for the significance of the difference in the mean between the baseline and Ketamine conditions across all 100 electrode assignments for the local and distributed clusters respectively. Significance is attained at the Bonferroni corrected threshold ($\alpha = 5*10^{-4}$) only for the distributed cluster in the α, β and γ, consistently across of cluster numbers. Similarly, tables Supplementary 7 and *Supplementary 8* show the same information for the difference in synchrony between the baseline and propofol conditions. Significance is attained at the Bonferroni corrected threshold ($\alpha = 5*10^{-4}$) only for the distributed cluster in the β and γ, consistently across of cluster numbers.

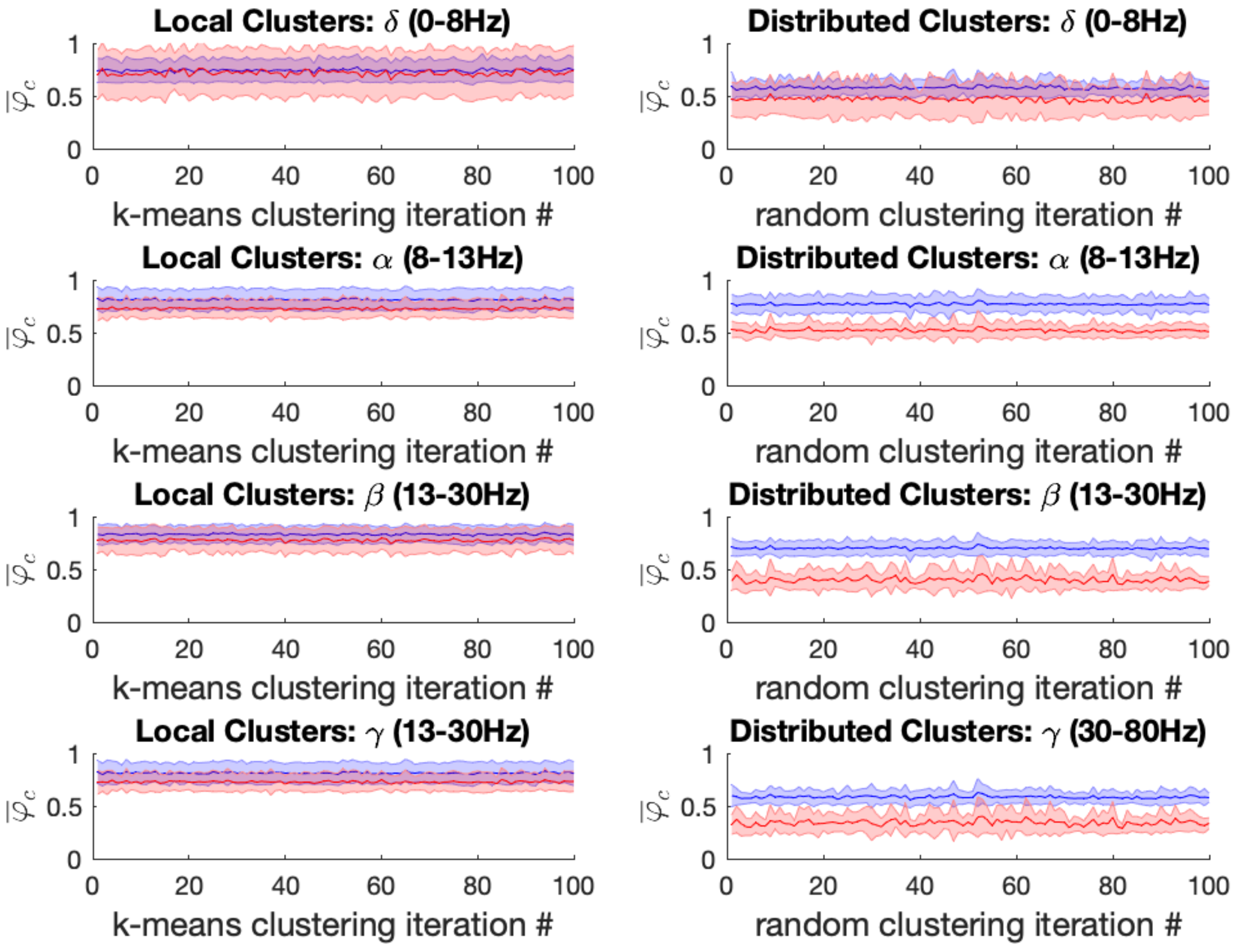

Supplementary 1 Left: For the ketamine (red) vs baseline (blue) conditions, the mean local cluster synchrony $\overline{\boldsymbol{\varphi}}_{\boldsymbol{c}} \pm$ standard deviation is plotted for each of 100 k-means iterations (x-axis) in each of the four frequency bands under study for k = 13. Repeated within-condition t-tests failed to reach statistical significance at the Bonferroni-corrected threshold in any of the four frequency bands of interest. Right: Mean distributed cluster synchrony $\overline{\boldsymbol{\varphi}}_{\boldsymbol{c}} \pm$ standard deviation is plotted for each of 100 iterations of the pseudo-random algorithm used for distributed cluster assignments (x-axis). Statistically significant reductions in distributed cluster synchrony at the Bonferroni-corrected significance threshold ($\alpha = 5*10^{-4}$) were observed following ketamine anesthesia for all (100 / 100) but

one distributed cluster assignments in the α, β and γ bands, respectively. By contrast no significant differences in drug effects on distributed cluster synchrony were observed in the δ-band.

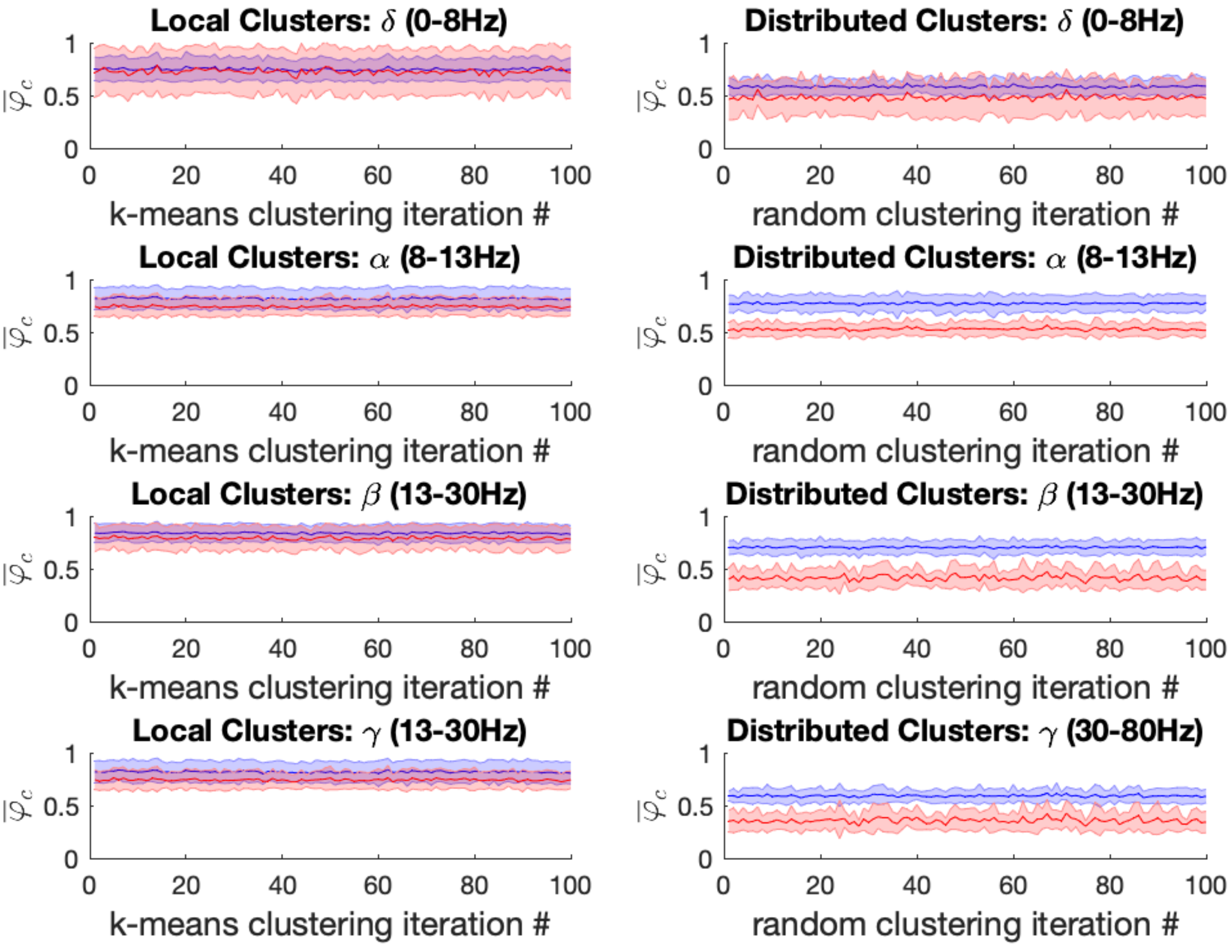

Supplementary 2 Left: For the ketamine (red) vs baseline (blue) conditions, the mean local cluster synchrony $\overline{\varphi}_c$ ± standard deviation is plotted for each of 100 k-means iterations (x-axis) in each of the four frequency bands under study for k = 15. Repeated within-condition t-tests failed to reach statistical significance at the Bonferroni-corrected threshold ($\alpha = 5*10^{-4}$) in any of the four frequency bands of interest. Right: Mean distributed cluster synchrony $\overline{\varphi}_c$ ± standard deviation is plotted for each of 100 iterations of the pseudo-random algorithm used for distributed cluster assignments (x-axis). Statistically significant reductions in distributed cluster synchrony at the Bonferroni-corrected significance threshold ($\alpha = 5*10^{-4}$) were observed following ketamine anesthesia for all (100 / 100)

distributed cluster assignments in the α, β and γ bands, respectively. By contrast no significant differences in drug effects on distributed cluster synchrony were observed in the δ-band.

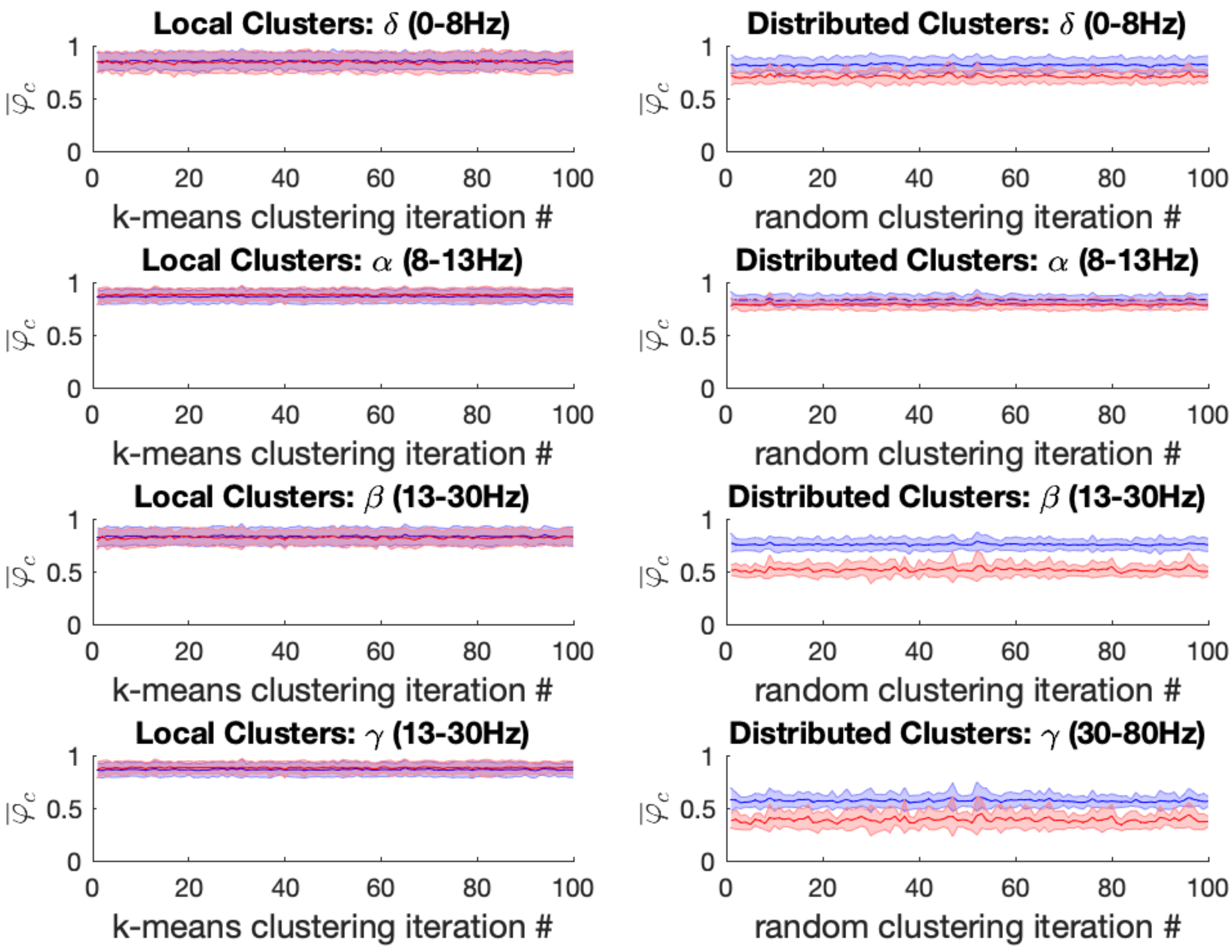

Supplementary 3 Left: For the propofol (red) vs baseline (blue) conditions, the mean local cluster synchrony $\overline{\boldsymbol{\varphi}}_{\boldsymbol{C}}$ ± standard deviation is plotted for each of 100 k-means iterations (x-axis) in each of the four frequency bands under study for k = 13. Repeated within-condition t-tests failed to reach statistical significance at the Bonferroni-corrected threshold (α = $5*10^{-4}$) in any of the four frequency bands of interest. Right: Mean distributed cluster synchrony $\overline{\boldsymbol{\varphi}}_{\boldsymbol{C}}$ ± standard deviation is plotted for each of 100 iterations of the pseudo-random algorithm used for distributed cluster assignments (x-axis). Statistically significant reductions in distributed cluster synchrony at the Bonferroni-corrected significance threshold (α = $5*10^{-4}$) were observed following propofol anesthesia for all (100/100) distributed cluster assignments in the β and γ bands, respectively. By contrast only 3/100 significant differences in

propofol's effect on distributed cluster synchrony were observed in the α-band, while more mixed results were observed in the δ-band with 58/100 of distributed cluster assignments reaching significance.

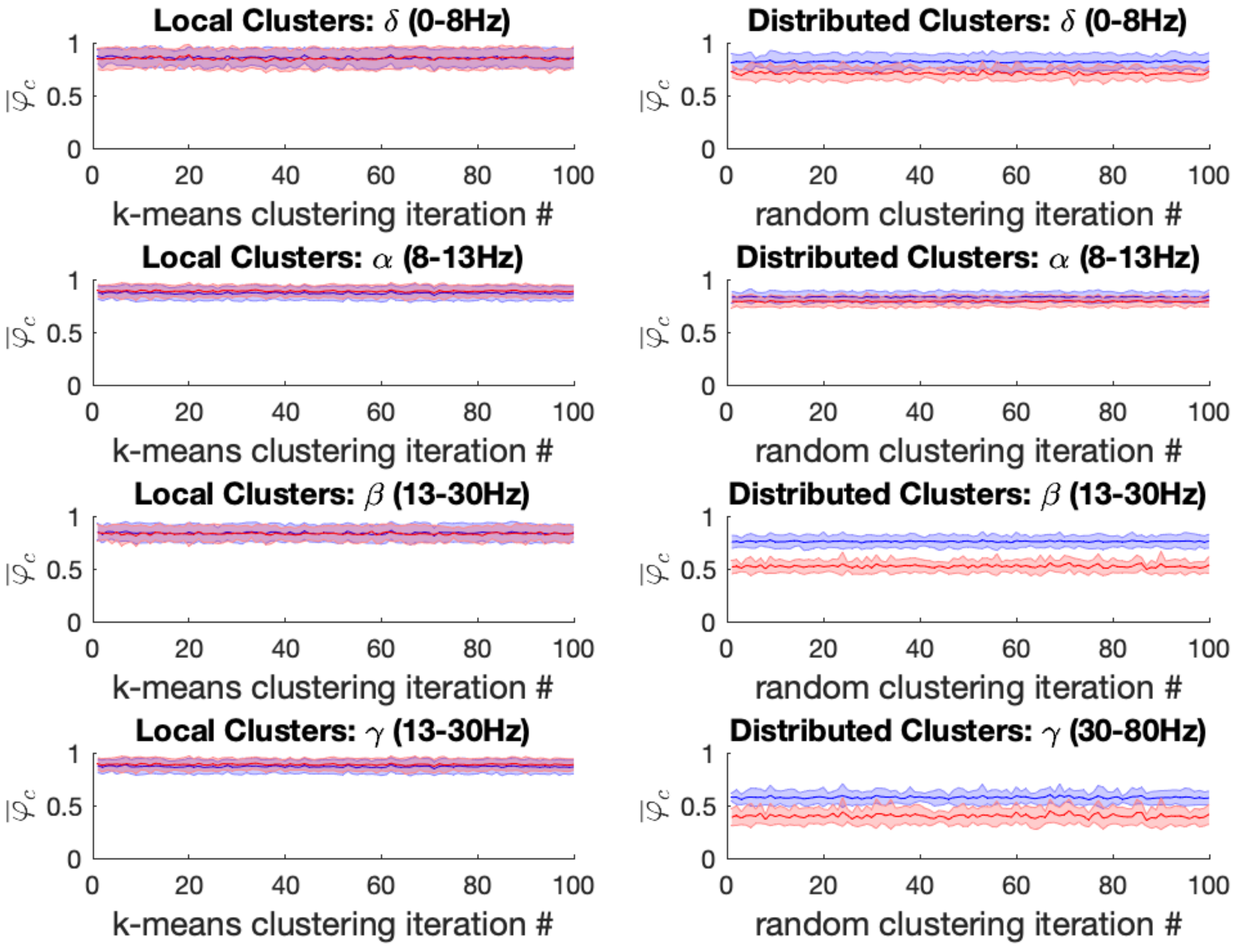

Supplementary 4 Left: For the propofol (red) vs baseline (blue) conditions, the mean local cluster synchrony $\overline{\boldsymbol{\varphi}}_{\boldsymbol{C}}$ ± standard deviation is plotted for each of 100 k-means iterations (x-axis) in each of the four frequency bands under study for k = 15. Repeated within-condition t-tests failed to reach statistical significance at the Bonferroni-corrected threshold ($\alpha = 5*10^{-4}$) in any of the four frequency bands of interest. Right: Mean distributed cluster synchrony $\overline{\boldsymbol{\varphi}}_{\boldsymbol{C}}$ ± standard deviation is plotted for each of 100 iterations of the pseudo-random algorithm used for distributed cluster assignments (x-axis). Statistically significant reductions in distributed cluster synchrony at the Bonferroni-corrected significance threshold ($\alpha = 5*10^{-4}$) were observed following propofol anesthesia for all (100/100) distributed cluster assignments in the β and γ bands, respectively. By contrast only 7/100 significant differences in propofol's effect on distributed cluster synchrony were observed in the α-band, while more mixed results were observed in the δ-band with 62/100 of distributed cluster assignments reaching significance.

	k=13	k=14	k=15
δ	0.634/(0/100)	0.618/(0/100)	0.641/(0/100)
α	0.013/(1/100)	0.010/(1/100)	0.013/(2/100)
β	0.097/(0/100)	0.019/(0/100)	0.119/(0/100)
γ	0.358/(0/100)	0.383/(0/100)	0.397/(0/100)

Supplementary 5 Average p-values for the mean local cluster synchrony between the baseline and Ketamine conditions for the four-frequency bands across the the 100 k-means iterations for k=13,14 and 15. Significance is attained for the α band in 1 or two trial, but the average p-values do not reach significance at the Bonferroni-corrected significance threshold ($\alpha = 5*10^{-4}$) .

	k=13	k=14	k=15
δ	0.087/(0/100)	0.09/(0/100)	0.093/(1/100)
α	$1*10^{-6}$/(100/100)	$3*10^{-7}$/(100/100)	$3*10^{-7}$/(100/100)
β	$3*10^{-6}$/(100/100)	$8*10^{-7}$/(100/100)	$3*10^{-6}$/(100/100)
γ	$1*10^{-5}$/(99/100)	$3*10^{-6}$/(100/100)	$2*10^{-6}$/(100/100)

Supplementary 6 Average p-values for the mean distributed cluster synchrony between the baseline and Ketamine conditions for the four-frequency bands across the 100 pseudo-random distributed class assignment algorithm iterations for k=13,14 and 15. Significance (in bold) is attained for the α, β and γ band at the Bonferroni-corrected significance threshold ($\alpha = 5*10^{-4}$) .

	k=13	k=14	k=15
δ	0.695/(0/100)	0.77/(0/100)	0.769/(0/100)
α	0.357/(0/100)	0.28/(0/100)	0.269/(0/100)

β	0.689/(0/100)	0.74/(0/100)	0.768/(0/100)
γ	0.816/(0/100)	0.76/(0/100)	0.744/(0/100)

Supplementary 7 Average p-values for the mean local cluster synchrony between the baseline and Propofol conditions for the four-frequency bands across the the 100 k-means iterations for k=13,14 and 15. Significance is never attained for any trial, and the average p-values do not reach significance at the Bonferroni-corrected significance threshold ($\alpha = 5*10^{-4}$).

	k=13	k=14	k=15
δ	0.006/(58/100)	0.001/(60/100)	0.003/(62/100)
α	0.022/(3/100)	0.017/(6/100)	0.018/(7/100)
β	$4*10^{-7}$/(100/100)	$1*10^{-7}$/(100/100)	$1*10^{-6}$/(100/100)
γ	$2*10^{-5}$/(100/100)	$2*10^{-5}$/(100/100)	$1*10^{-5}$/(100/100)

Supplementary 8 Average p-values for the mean distributed cluster synchrony between the baseline and Propofol conditions for the four-frequency bands across the 100 pseudo-random distributed class assignment algorithm iterations for k=13,14 and 15. Significance (in bold) is attained for the β and γ band at the Bonferroni-corrected significance threshold ($\alpha = 5*10^{-4}$).
